# Ascorbate-Mediated Modulation of Cadmium Stress Responses: Reactive Oxygen Species and Redox Status in *Brassica napus*

**DOI:** 10.3389/fpls.2020.586547

**Published:** 2020-11-30

**Authors:** Ha-il Jung, Bok-Rye Lee, Mi-Jin Chae, Eun-Jin Lee, Tae-Gu Lee, Goo-Bok Jung, Myung-Sook Kim, Jinwook Lee

**Affiliations:** ^1^Division of Soil and Fertilizer, National Institute of Agricultural Sciences, Rural Development Administration, Wanju, South Korea; ^2^Department of Animal Science, Institute of Agricultural Science and Technology, College of Agriculture and Life Science, Chonnam National University, Gwangju, South Korea; ^3^Division of Climate Change and Agroecology, National Institute of Agricultural Sciences, Rural Development Administration, Wanju, South Korea; ^4^Department of Plant Science and Technology, Chung-Ang University, Anseong, South Korea

**Keywords:** ascorbate, ascorbate–glutathione–NADPH cycle, cadmium toxicity, oilseed rape, reactive oxygen species

## Abstract

The role of ascorbate (AsA) in antioxidant defense system-associated resistance to cadmium (Cd) in oilseed rape plants has not yet been clearly demonstrated. The present study investigated the critical role of exogenous AsA on the physiological and biochemical responses of reactive oxygen species (ROS) and antioxidant scavenging defense systems in oilseed rape (*Brassica napus* L. cv. Tammi) seedlings exposed to Cd. Cd (10 μM) treatment led to significant reductions in plant growth; increases in the levels of superoxide anion radical, hydrogen peroxide, and malondialdehyde; and increases in Cd uptake and accumulation by the roots and shoots in hydroponically grown 10-day-old seedlings. Moreover, it reduced AsA content and AsA redox ratios, which have been correlated with reductions in glutathione (GSH) and/or nicotinamide adenine dinucleotide phosphate (NADPH) redox status. However, exogenously applying AsA to Cd-exposed seedlings decreased Cd-induced ROS, improved antioxidant defense systems by increasing AsA, GSH, and NADPH contents, and increased Cd uptake and accumulation in both roots and shoots of the plants. These results provided evidence that the enhancement in AsA redox status can be linked to an increase in the GSH and/or NADPH redox ratios through the induction of the AsA–GSH–NADPH cycle. Thus, these results suggest that exogenous AsA application to oilseed rape seedlings under Cd stress might alleviate the overall Cd toxicity by regulating the homeostasis of the AsA–GSH–NADPH cycle, which reestablishes the steady-state cellular redox status.

## Introduction

Cadmium (Cd) pollution is rapidly increasing because of global urbanization and industrialization, whereas the concomitant increase in contaminated farmland and agricultural water supplies has serious effects on the safety of agricultural production. Moreover, because Cd is highly mobile in soil, it readily contaminates agricultural products through uptake and translocation, even in crops cultivated in soil containing low concentrations of Cd. Thus, Cd is a factor that threatens human health, as humans are high trophic level consumers and may experience Cd biomagnification (Kim et al., 2008; Hong et al., 2013; Jan et al., 2015; Khan et al., 2015; Jung et al., 2016, 2018b; Farooq et al., 2018).

By weakening the affinity of sulfhydryl groups (-SH) in proteins and disrupting the activity of metabolically important enzymes, Cd can cause fatal impairments in both plants and humans (Meuwly and Rauser, 1992; Jung et al., 2015, 2016, 2018b). In addition to inhibiting normal plant growth, Cd can replace the cofactors of essential metal ions in important proteins through the Fenton reaction ([Bibr B45][Bibr B24]; Jung et al., 2016; Singh et al., 2016; Loix et al., 2017; Jung et al., 2018b). This process generates reactive oxygen species (ROS), such as the superoxide anion radical (O_2_^⋅–^), hydrogen peroxide (H_2_O_2_), hydroxyl radicals (OH⋅), and so on. ROS not only destroy cell membranes by intracellular lipid peroxidation but also decrease the biosynthesis of primary metabolites required for plant life, such as carbohydrates, proteins, fats, and nucleic acids, and significantly impair normal vital functions by adversely affecting photosynthesis, respiration, ion absorption, oxidation/reduction, and the homeostatic balance of hormones (Gayomba et al., 2013; Jung et al., 2015, 2016; Singh et al., 2016; Asgari Lajayer et al., 2017). Cd toxicity can be counteracted using various physiological and biochemical detoxification systems to alleviate or minimize oxidative stress caused by excessive absorption and accumulation of Cd (Kim et al., 2008; Srivastava et al., 2014; Singh et al., 2016; Jung et al., 2017, 2018b).

Two antioxidant defense pathways can be used to eliminate the oxidative stress caused by Cd, thereby contributing to maintaining redox homeostasis. The first is an enzymatic antioxidant scavenging pathway using antioxidant or redox enzymes, such as superoxide dismutase (SOD), catalase (CAT), ascorbate peroxidase (APX), monodehydroascorbate reductase (MDHAR), dehydroascorbate reductase (DHAR), and glutathione reductase (GR); the second is a non-enzymatic antioxidant scavenging pathway that uses antioxidant or redox metabolites, including ascorbate (AsA), glutathione (GSH), and nicotinamide adenine dinucleotide phosphate (NADPH) to relieve excessive accumulation of ROS (Gill and Tuteja, 2010; Shekhawat et al., 2010; Foyer and Noctor, 2011; Yan et al., 2015; Sharma et al., 2017).

The roles and functions of antioxidant enzymes and non-enzymatic GSH in scavenging the ROS produced by heavy metals have been thoroughly investigated through numerous studies in various plants (Srivastava et al., 2014; Singh et al., 2015). However, studies on the alleviation of oxidative stress caused by heavy metals through the application of AsA, which plays a critical role in ROS scavenging, redox regulation, plant cell cycle, plant cell growth and development, and damaged DNA restoration, are limited (Akram et al., 2017; Jung et al., 2018a). Akram et al. (2017) comprehensively and systematically demonstrated that AsA plays a very important role in reducing oxidative stress by reviewing the previous reported studies that examined the effect of AsA application on numerous plants subjected to various environmental abiotic stressors including high and low temperature, drought, and salinity. Despite the fact that AsA is used in many different fields to mitigate oxidative stress induced by various environmental stressors (Akram et al., 2017; Chen et al., 2017), there are only a few studies reported on the interaction between Cd toxicity and AsA (Chao et al., 2010; Semida et al., 2018). Chao et al. (2010) reported that hydroponic treatment with AsA reduces malondialdehyde (MDA) production and increases chlorophyll content, thereby contributing to reversing the effects of Cd toxicity in rice. Recently, Semida et al. (2018) reported that sequentially treating seeds with AsA-proline-GSH reduced Cd toxicity in cucumber seedlings and increased plant heavy metal tolerance. Moreover, Jung et al. (2018a) and Zhang et al. (2019) demonstrated the effects of AsA, as a component of the antioxidant-scavenging and redox-regulating system, on reducing arsenic (As) and Cd toxicity in rice and maize plants, respectively.

Cd contamination not only results in Cd toxicity to plants but also negatively affects the uptake of plant macronutrients (Khan et al., 2015; Jung et al., 2016, 2018b). By inhibiting the absorption of essential macro-elements, Cd causes overall disruption of plant physiological and metabolic activities and impairs normal plant growth and development (Asgari Lajayer et al., 2017). Therefore, understanding and elucidating the effects of AsA on macro-element absorption in plants experiencing Cd stress will not only provide basic information about its influence on plant macro-element absorption but also inform us regarding its potential to alleviate the antagonistic influences of Cd on nutrient assimilation.

Oilseed rape (*Brassica napus* L.) belongs to the family Brassicaceae, which is grown worldwide as an oilseed crop. Plants of Brassicaceae have been used as potential candidates for phytoextraction of heavy metals including Cd (Gall and Rajakaruna, 2013; Ali et al., 2014; Ahmad et al., 2015). *B. napus* stands out as a Cd hyperaccumulator owing to its high Cd-accumulating ability, fast plant growth and development, and high biomass in comparison to other natural metal accumulators (Meng et al., 2009; Hasanuzzaman et al., 2017). Nevertheless, AsA-mediated detoxification has not been studied in oilseed rape under Cd stress. Thus, this study was conducted to elucidate the mechanisms whereby Cd phytotoxicity in oilseed rape may be alleviated. To accomplish this, we evaluated the effects of AsA on Cd uptake, Cd accumulation, ROS production, and redox homeostasis, all of which are related to Cd-induced phytotoxicity, in oilseed rape grown under Cd-contaminated conditions. We also examined the effectiveness of lower-cost foliar (as opposed to soil) AsA treatments to mitigate Cd phytotoxicity in a Cd hyperaccumulator plant (*B. napus*) that is a candidate for Cd phytoremediation while also simultaneously examining the mechanisms through which AsA provides Cd resistance. Therefore, the objective of this study was to test the hypothesis that the foliar application of exogenous AsA would be involved in reducing the phytotoxicity caused by the excessive uptake and accumulation of Cd, as well as evaluating the effects on ROS and redox homeostasis, and elucidating whether AsA can be used to remediate Cd-contaminated soil using plants, especially oilseed rape.

## Materials and Methods

### Plant Cultivation and Experimental Design

Oilseed rape (*Brassica napus* L. cv. Tammi) seeds were surface-sterilized with 70% ethanol for 3 min and rinsed extensively with distilled water. The surface-sterilized seeds were germinated under moist conditions (i.e., seeds were covered with two paper towel layers moistened with distilled water) at 25°C for 48 h. The germinated seeds were transferred to a hydroponic growth system containing a hydroponic solution. Uniform three-leaf-stage seedlings were transplanted into plastic pots (two plants per pot) for cultivation. These pots (30 cm × 25 cm × 15 cm, 0.075-m^2^ surface area) were filled with 9 L of hydroponic solution containing (mM for the macro-elements): 1.5 NH_4_NO_3_, 0.505 KH_2_PO_4_, 0.045 K_2_HPO_4_, 1 K_2_SO_4_, 3 CaCl_2_, 0.5 MgSO_4_, and 0.4 Fe-EDTA and (μM for the micro-elements): 14 H_3_BO_3_, 5 MnCl_2_⋅4H_2_O, 0.7 CuSO_4_⋅5H_2_O, 3 ZnSO_4_⋅7H_2_O, 0.7 (NH_4_)_6_Mo_7_O_24_⋅4H_2_O, and 0.1 CoCl_2_⋅6H_2_O. The hydroponic solution was pH adjusted to 5.6, continuously aerated, and replaced every 5 days (Lee et al., 2016). The hydroponically cultivated seedlings were grown in a greenhouse (National Institute of Agricultural Sciences, RDA, Wanju, Jeollabuk-do, Korea) with natural sunlight, day/night temperatures of 27°C/22°C, and day/night relative humidity of 60%/80%. Plastic pots for each treatment were rearranged daily during the growth and treatment period to minimize environmental variation.

We established five treatments with three replicates per treatment: Cd only, Cd + AsA (250 mg kg^–1^), Cd + AsA (500 mg kg^–1^), AsA only, and an untreated control (no Cd, no AsA); however, we do not present results for the AsA-only treatment as a positive control in this manuscript. According to the previous research results (Chao et al., 2010; Xu et al., 2015; Jung et al., 2018a), the phenotypic responses were not different by AsA-only treatment compared with untreated control treatment. To accomplish this, five-leaf seedlings were grown in hydroponics treated with 10 μM Cd (CdCl_2_) and were simultaneously sprayed with 250 mg kg^–1^ (1.42 mM) and 500 mg kg^–1^ (2.84 mM) AsA and 2 ml L^–1^ commercial surfactant (10% polyoxyethylene alkyl aryl ether and 20% sodium lignosulfonate). AsA was applied just once using a handheld sprayer delivering 1,000 L ha^–1^ of solution *via* flat-fan spray tips (Jung et al., 2019). Ten days after treatment, oilseed rape seedlings were removed from pots and tapped with a paper towel to remove water. Then, plant growth characteristics were determined correspondingly. Subsamples (200 mg) of roots and leaves were flash-frozen in liquid nitrogen and stored at −80°C until the ROS and redox analyses.

### Plant Growth Response

The effect of AsA application on the plant growth characteristics of oilseed rape seedlings grown in Cd-treated hydroponics with or without the AsA was estimated by determining shoot fresh weight (FW), shoot dry weight (DW), root DW, and shoot water content (WC). After the 10-day treatment period, the seedlings were separated into roots and leaves, and the shoot FW was determined. For shoot WC measurement, the shoots were excised, and their FW was immediately recorded. For shoot DW measurement, the weighed fresh shoots were oven-dried at 60°C for 72 h and then weighed again. Shoot WC in oilseed rape plant was calculated as follows: Shoot WC (%) = (Shoot FW – Shoot DW)/Shoot FW × 100.

### Determination of Cadmium and Macro-Mineral Concentrations

Oilseed rape root and shoot samples were first thoroughly washed with tap water and rinsed extensively with distilled water. Then, the samples were dried at 80°C for 72 h in an oven and subsequently powdered. For determining Cd and macro-mineral contents in roots and shoots of oilseed rape plants, 200 mg of the powder sample was digested using the Graphite Block Acid Digestion System (ODLAB Co., Ltd., Seoul, Korea). After digestion, the solutions were cooled to ambient temperature, diluted to 100 ml with ultrapure water, and filtered through Grade No. 40 filter papers (Whatman Co., Buckinghamshire, United Kingdom). Cd and macro-mineral contents using this filtrate were determined *via* inductively coupled plasma-mass spectroscopy (ICP-MS) analysis (Agilent 7900; Agilent Technologies Inc., Santa Clara, CA, United States). The following macro-minerals were analyzed: magnesium (Mg), calcium (Ca), iron (Fe), and potassium (K). Cd accumulation in oilseed rape organs was calculated as follows: Cd accumulation = *Ca* × *Cb*, where *Ca* is the Cd concentration and *Cb* is the dry weight in the root and shoot of an oilseed rape plant.

### Determination of Superoxide, Hydrogen Peroxide, and Malondialdehyde

Superoxide anion radical (O_2_^⋅–^) content was determined spectroscopically at 530 nm, following the method developed by Elstner and Heupel (1976); a standard NaNO_2_ curve was used for calculations. H_2_O_2_⋅ content was measured colorimetrically according to the method described by Jana and Choudhuri (1982). Sample absorbance was measured at 410 nm, and H_2_O_2_ content was calculated using an extinction coefficient of 0.28 μM^–1^ cm^–1^. Lipid peroxidation was indirectly estimated using the level of MDA by the thiobarbituric acid method of Buege and Aust (1978) with slight modifications. Sample absorbance was measured at 532 nm and corrected for non-specific turbidity by subtracting the value from the absorbance at 600 nm. MDA content was calculated using an extinction coefficient of 156 mM^–1^ cm^–1^.

### Determination of AsA/DHA, GSH/GSSG, and NADPH/NADP^+^

The total AsA, reduced AsA, and oxidized dehydroascorbate (DHA) contents were assayed colorimetrically according to the method described by Law et al. (1983). The absorbance of samples was calculated at 525 nm, and the total and reduced AsA contents were calculated using a standard AsA curve. The DHA content was recalculated by subtracting the reduced AsA content from the total AsA content.

The reduced GSH and oxidized GSSG contents were assayed according to the method described by Meister and Anderson (1983). The absorbance of samples was calculated at 412 nm for 1 min, and total GSH and oxidized GSSG contents were determined using linear regression obtained from a standard curve of GSH and GSSG. The reduced GSH content was recalculated by subtracting the oxidized GSSG content from the total GSH content.

The reduced NADPH and oxidized NADP^+^ contents were assayed following the method of Queval and Noctor (2007). For the NADPH and NADP^+^ extraction, frozen root and leaf organs were ground in liquid nitrogen and then resuspended with either 0.8 ml of 0.2 M HCl or 0.2 M NaOH, respectively. One hundred microliters per extract was heated at 95°C for 1 min and stopped in an ice bath. The supernatant was neutralized by 0.2 M HCl to a final pH of 7–8 for the NADPH assay and counteracted by 0.2 M NaOH to a final pH of 5–6 for the NADP^+^ assay. Forty microliters were added to each reaction mixture containing 0.1 M HEPES (pH 7.5) that consisted of 2 mM Na_2_EDTA, 1.2 mM dichlorophenolindophenol (DCPIP), 20 mM phenazine methosulfate (PMS), and 10 mM glucose-6-phosphate. The reaction started by adding 2 μl glucose 6-phosphate dehydrogenase (G6PDH, 200U). The NADPH and NADP^+^ contents were determined using a standard curve with standards of 1–100 pmol.

### Determination of Antioxidant Enzyme Activities

The antioxidant enzymes were extracted with 100 mM potassium phosphate buffer (pH 7.5) containing 2 mM of ethylenediaminetetraacetic acid (EDTA), 1% polyvinyl pyrrolidone-40 (PVP-40), and 1 mM of phenylmethylsulfonyl fluoride (PMSF), as described by Lee et al. (2013). After enzyme extraction of samples, the protein concentration was determined using the detailed method of Kruger (1994).

SOD (EC 1.15.1.1) activity was assayed according to Lee et al. (2009) by measuring the enzyme ability to inhibit the photoreduction of nitroblue tetrazolium (NBT). One unit of SOD activity was defined as the amount of enzyme that inhibited the rate of NBT photoreduction by 50% at 560 nm. SOD activity indicated units of SOD mg^–1^ protein.

CAT (EC 1.11.1.6) activity was determined based on the method of Lee et al. (2013) by monitoring the decrease in absorbance at 240 nm for 1 min (extinct coefficient, ε = 36 mM^–1^ cm^–1^) as a result of H_2_O_2_ degradation. CAT activity indicated μmol H_2_O_2_ decomposed min^–1^ mg^–1^ protein.

APX (EC 1.11.1.11) activity was measured according to the method of Chen and Asada (1989) by monitoring the decline in absorbance at 290 nm for 1 min (extinct coefficient, ε = 2.8 mM^–1^ cm^–1^) as AsA was oxidized. APX activity indicated μmol ascorbate min^–1^ mg^–1^ protein.

MDHAR (EC 1.6.5.4) activity was evaluated using the method described by Hossain et al. (1984) by monitoring the decrease in absorbance at 340 nm for 1 min (6.2 mM^–1^ cm^–1^) as NADPH was oxidized. MDHAR activity indicated nmol NADH min^–1^ mg^–1^ protein.

DHAR (EC 2.5.1.1.8) activity was measured colorimetrically according to the method described by Nakano and Asada (1981) by monitoring the increase in absorbance at 265 nm for 1 min (14 mM^–1^ cm^–1^) as DHA was reduced. DHAR activity indicated nmol ascorbate min^–1^ mg^–1^ protein.

GR (EC 1.6.4.2) activity was assayed according to the method of Rao et al. (1996) by monitoring the decrease in absorbance at 340 nm for 1 min (extinct coefficient, ε = 6.2 mM^–1^ cm^–1^) as NADPH was oxidized. GR activity indicated μmol NADPH min^–1^ mg^–1^ protein.

### Statistical Analysis

Analysis of variance (ANOVA) was applied to all data, and Fisher’s least significant difference (LSD) test was used to determine significant differences among treatments. All statistical analyses were performed using statistical analysis software (SAS ver. 9.2; SAS Institute Inc., Cary, NC, United States). Differences at *P* < 0.05 were considered significant. In the figures, the data are expressed as mean ± standard deviation (SD).

To elucidate the response of targeted metabolites of ROS and AsA/GSH/NADPH redox status in the roots and leaves of oilseed rape plants treated with different levels of AsA, correlation coefficient analysis and principal component analysis (PCA) were performed using MetaboAnalyst 4.0^1^ (Chong et al., 2018) for heatmap generation and cluster analysis, respectively.

## Results

### Changes in the Plant Growth Characteristics in Cadmium-Stressed Oilseed Rape Seedlings With or Without Ascorbate Application

Shoot FW and DW of the Cd-only-treated seedlings were significantly decreased by Cd treatment compared with those of the untreated plants. However, Cd treatment had no effect on the root DW and shoot WC. Moreover, plant growth characteristics were not significantly different in the untreated seedlings than in the seedlings treated with AsA alone (data not shown). Notably, exogenous applications of AsA to Cd-exposed seedlings significantly alleviated the negative influences of Cd on the shoot FW and DW of these plants (Table 1).

**TABLE 1:** Effect of ascorbate (AsA) application on plant growth characteristics of oilseed rape seedlings grown in cadmium (Cd)-treated hydroponics.

**Treatment**	**Shoot FW (g)**	**Shoot DW (g)**	**Root DW (g)**	**Shoot WC (%)**
Control	64.5 ± 0.9 a	4.73 ± 0.20 a	0.33 ± 0.03 a	92.7 ± 0.4 a
10 μM Cd + 0 mg AsA kg^–1^	54.7 ± 2.2 b	3.91 ± 0.18 b	0.30 ± 0.05 a	92.6 ± 0.7 a
10 μM Cd + 250 mg AsA kg^–1^	62.8 ± 0.8 a	4.60 ± 0.03 a	0.32 ± 0.02 a	92.8 ± 0.2 a
10 μM Cd + 500 mg AsA kg^–1^	64.7 ± 3.8 a	4.70 ± 0.06 a	0.33 ± 0.02 a	92.6 ± 0.4 a

*Oilseed rape seedlings at the five-leaf stage were cultivated in a hydroponic solution containing 10 μM Cd and sprayed with AsA (250 and 500 mg kg^–1^). The shoot fresh weight (FW), shoot dry weight (DW), root DW, and shoot water content (WC) were measured at 10 days after treatment. ^†^Means within a column followed by the same letter are not significantly different at the *P* < 0.05 level based on Fisher’s least significant difference (LSD) tests.*

### Changes in the Cadmium Uptake and Accumulation in Cadmium-Stressed Oilseed Rape Seedlings With or Without Ascorbate Application

**FIGURE 1:**
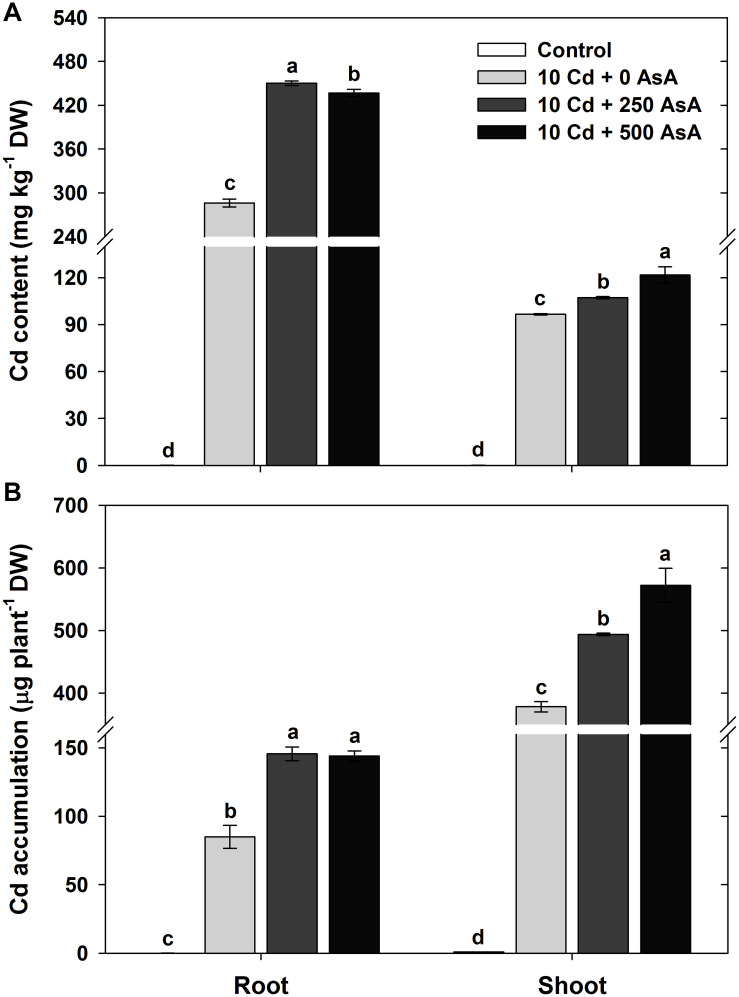
Effect of ascorbate (AsA) application on the cadmium (Cd) content **(A)** and Cd accumulation **(B)** of the root and shoot of oilseed rape seedlings grown in Cd-treated hydroponics. Oilseed rape seedlings at the five-leaf stage were cultivated in a hydroponic solution containing 10 μM Cd and sprayed with AsA (250 and 500 mg kg^–1^). Ten days after treatment, the level of Cd in the roots and shoots was measured *via* inductively coupled plasma-mass spectroscopy analysis. The data are presented as mean ± standard deviation (SD) of the mean of three replications (*n* = 3). Means denoted by the same letter are not significantly different at the *P* < 0.05 level according to Fisher’s least significant difference (LSD) test.

In the oilseed rape seedlings treated with Cd alone, the Cd content was significantly higher in the roots than that in the shoots. Application of 250 and 500 mg kg^–1^ AsA in Cd-treated plants enhanced Cd uptake and accumulation. AsA treatment induced approximately 1.57- and 1.11-fold increases in Cd uptake in the roots and shoots, respectively, relative to Cd uptake in roots and shoots of plants treated with Cd alone (Figure 1A). Although the evaluation of Cd accumulation in the roots and shoots was based on the biomass (root and shoot DW per plant), trends for Cd accumulation were similar to those for Cd uptake. The Cd accumulation was 1.72- and 1.70-fold higher in the roots and 1.31- and 1.51-fold higher in the shoots in the 250 and 500 mg kg^–1^ AsA treatments, respectively, than that in the Cd-only treatment (Figure 1B).

### Changes in the Macro-Mineral Uptake in Cadmium-Stressed Oilseed Rape Seedlings With or Without Ascorbate Application

**FIGURE 2:**
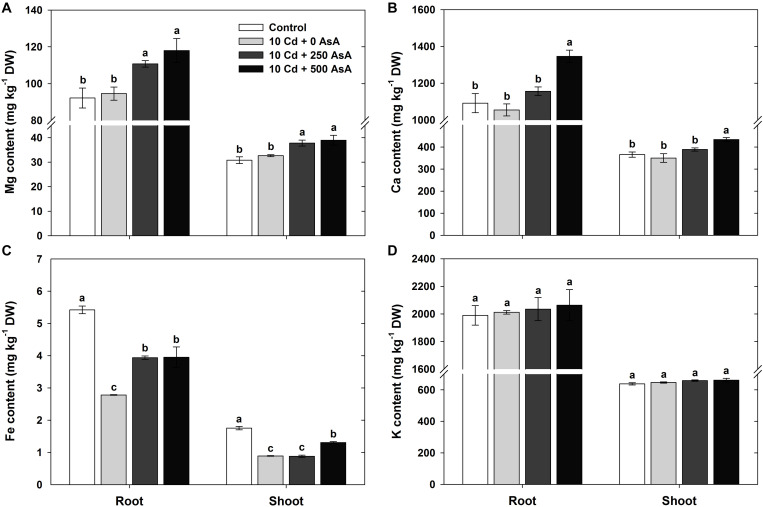
Effect of ascorbate (AsA) application on the magnesium (Mg) **(A)**, calcium (Ca) **(B)**, iron (Fe) **(C)**, and potassium (K) **(D)** contents of the root and shoot of oilseed rape seedlings grown in cadmium (Cd)-treated hydroponics. Oilseed rape seedlings at the five-leaf stage were cultivated in a hydroponic solution containing 10 μM Cd and sprayed with AsA (250 and 500 mg kg^–1^). Ten days after treatment, the level of each cation in the roots and shoots was measured *via* inductively coupled plasma-mass spectroscopy analysis. The data are presented as mean ± standard deviation (SD) of the mean of three replications (*n* = 3). Means denoted by the same letter are not significantly different at the *P* < 0.05 level according to Fisher’s least significant difference (LSD) test.

The macro-minerals Mg, Ca, Fe, and K were measured in the roots and shoots to evaluate the effect of AsA in the Cd-stressed seedlings on nutrient acquisition. Compared with that of the untreated plants, Cd treatment did not inhibit Mg, Ca, and K uptake in the roots and shoots (Figures 2A,B,D). However, the Fe content in both plant organs was significantly reduced by Cd treatment (Figure 2C). Notably, the plants subjected to AsA treatments exhibited significantly higher contents of Mg, Ca, and Fe in the roots and shoots than those treated with Cd alone (Figures 2A–C).

### Reactive Oxygen Species Production in Cadmium-Stressed Oilseed Rape Seedlings With or Without Ascorbate Application

**FIGURE 3:**
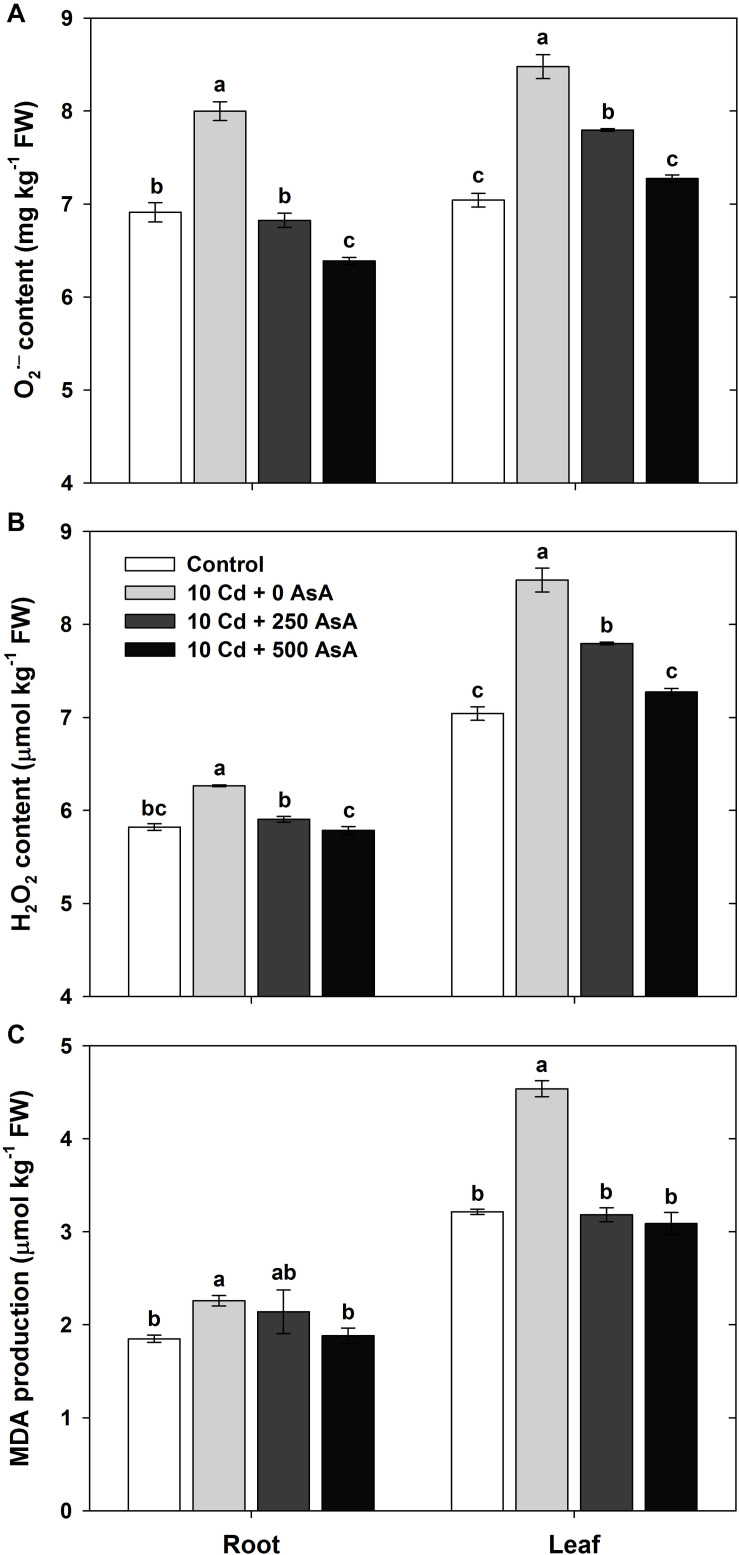
Effect of ascorbate (AsA) application on the contents of superoxide [**(A)** O_2_^⋅–^)] and hydrogen peroxide [**(B)**; H_2_O_2_] and malondialdehyde (MDA) production [**(C)** MDA] of oilseed rape seedlings grown in cadmium (Cd)-treated hydroponics. Oilseed rape seedlings at the five-leaf stage were cultivated in a hydroponic solution containing 10 μM Cd and sprayed with AsA (250 and 500 mg kg^–1^). Ten days after treatment, the contents of O_2_^⋅–^, H_2_O_2_, and MDA in the roots and leaves were measured. The data are presented as mean ± standard deviation (SD) of the mean of three replications (*n* = 3). Means denoted by the same letter are not significantly different at the *P* < 0.05 level according to Fisher’s least significant difference (LSD) test.

To quantify the oxidative responses of oilseed rape seedling to Cd toxicity, we determined the O_2_^⋅–^ (Figure 3A), H_2_O_2_ (Figure 3B), and MDA (Figure 3C) contents in plants at 10 days after AsA application and Cd treatment. First, we note that ROS production in oilseed rape seedlings that were treated only with AsA (no addition of Cd) was not significantly different from ROS production in the untreated control plants (data not shown).

Cd stress increased ROS production in *B. napus* seedlings. The contents of each of these compounds in both organs were significantly higher in Cd-exposed plants than those in untreated seedlings. Oilseed rape plants grown in the Cd-treated hydroponics showed severe symptoms of Cd toxicity, such as plant growth inhibition, compared with those of the untreated control (Table 1).

However, exogenous applications of 250 and 500 mg kg^–1^ AsA to Cd-treated seedlings significantly reduced O_2_^⋅–^ and H_2_O_2_ content compared to that of the plants treated with Cd alone; thus, AsA application rescued seedlings from the increased ROS production induced by Cd toxicity (Figures 3A,B). In addition, AsA application significantly decreased the MDA content produced by Cd toxicity in both organs (Figure 3C).

### Redox Status in Cadmium-Stressed Oilseed Rape Seedlings

When plants are exposed to Cd stress, the alteration of the cellular redox conditions could be closely associated not only with the AsA redox ratio (AsA/DHA) and the GSH redox ratio (GSH/GSSG), but also with the NADPH redox ratio (NADPH/NADP^+^). To understand the effect of Cd stress on plant redox status, the AsA, GSH, and NADPH levels and redox status in roots and leaves were evaluated at 10 days after plants had either received Cd or no treatment. All comparisons below are between Cd-treated (no AsA) seedlings and the untreated control seedlings.

**FIGURE 4:**
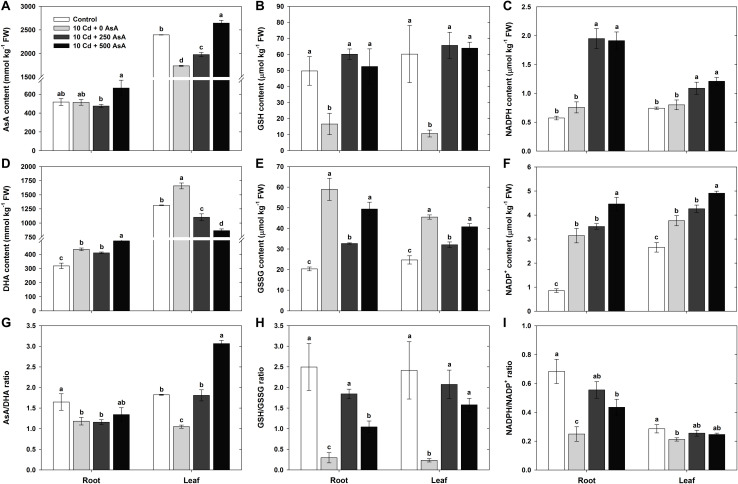
Effect of ascorbate (AsA) application on the contents of AsA (reduced form)/glutathione (GSH, reduced form)/nicotinamide adenine dinucleotide phosphate (NADPH, reduced form) and dehydroascorbate (DHA, oxidized form)/GSH disulfide (GSSG, oxidized form)/nicotinamide adenine dinucleotide phosphate (NADP^+^, oxidized form) and the AsA/GSH/NADPH redox ratio in oilseed rape seedlings grown in the cadmium (Cd)-treated hydroponics. Oilseed rape seedlings at the five-leaf stage were cultivated in a hydroponic solution containing 10 μM Cd and sprayed with AsA (250 and 500 mg kg^–1^). Ten days after treatment, the levels of AsA/GSH/NADPH and DHA/GSSG/NADP^+^ and the AsA/GSH/NADPH redox ratio in the roots and leaves were measured. **(A–C)** Content of AsA, GSH, and NADPH. **(D–F)** Contents of DHA, GSSG, and NADP^+^. **(G–I)** AsA, GSH, and NADPH redox ratios (AsA/DHA, GSH/GSSG, and NADPH/NADP^+^). The data are presented as mean ± standard deviation (SD) of the mean of three replications (*n* = 3). Means denoted by the same letter are not significantly different at the *P* < 0.05 level according to Fisher’s least significant difference (LSD) test.

Cd toxicity increased both the DHA level and the AsA redox status in *B. napus* seedlings, but it had differential plant organ-specific effects on AsA levels. In Cd-stressed seedlings, the AsA level in roots was not significantly different from that of the untreated control seedlings, but leaf AsA levels declined significantly (Figure 4A). However, the DHA level, the oxidized form of AsA, was significantly higher in both roots (1.37-fold increase) and leaves (1.26-fold increase) of plants exposed to Cd (Figure 4D). Cd treatment reduced AsA redox status in both roots (−28%) and leaves (−42%) compared to those of the untreated control plants (Figure 4G).

Cd toxicity increased GSSG but reduced GSH and the GSH redox status in *B. napus* seedlings. The GSH level in the roots and leaves of Cd-treated plants was 67 and 82% lower than that of the untreated plants, respectively (Figure 4B). However, the level of GSSG, the oxidized form of GSH, in roots and leaves was 290 and 184% higher in the Cd-stressed seedlings than that in the untreated seedlings, respectively (Figure 4E). The GSH redox status in the Cd-stressed seedlings greatly decreased by 88% in the roots and by 90% in the leaves compared to those of the untreated control plants (Figure 4H).

Finally, Cd toxicity increased NADPH, reduced NADPH redox ratios, and had no effect on NADP^+^ levels in *B. napus* seedlings. The NADPH contents of Cd-treated seedling roots and leaves were not statistically different from those of the untreated controls (Figure 4C). In contrast, the content of NADP^+^, the oxidized form of NADPH, largely increased in the roots and leaves under Cd stress (Figure 4F). NADPH redox ratios were significantly lower in the roots and leaves of the Cd-stressed plants than those in the untreated plants (Figure 4I).

### Redox Status in Cadmium-Stressed Oilseed Rape Treated With Ascorbate

In addition to examining the effects of Cd stress on seedling redox status, we also examined the effects of applying AsA to plants experiencing Cd stress and compared them with plants treated with Cd. To evaluate the potential of AsA to ameliorate the effects of Cd stress on seedling redox status, the AsA, GSH, and NADPH levels and redox status in roots and leaves were evaluated at 10 days posttreatment with Cd and/or AsA. All comparisons below are between seedlings receiving Cd + AsA and seedlings receiving only Cd. Moreover, two levels of AsA application were evaluated: 250 and AsA 500 mg kg^–1^.

Applying AsA to Cd-stressed plants increased their AsA content across both seedling plant organ types (i.e., leaves and roots) and AsA treatment levels (i.e., 250 and 500 mg kg^–1^), but the effects of AsA application on DHA and AsA redox ratios varied depending on the plant organ type and the amount of AsA applied. The AsA content increased in the roots and leaves of AsA-treated plants (Cd + AsA) compared to that of Cd-only-treated plants (Figure 4A). On the other hand, root DHA content did not change with the application of 250 mg kg^–1^ AsA and slightly increased in response to 500 mg kg^–1^ AsA, but leaf DHA significantly decreased in both AsA treatments (Figure 4D). The AsA redox ratio in roots was not significantly different at either level of AsA treatment from that in the Cd-only treatment (Figure 4G). However, a high AsA redox ratio was maintained in the leaves because AsA application had both increased the AsA content and decreased the DHA content relative to the Cd-only-treated plants (Figure 4G).

AsA application dramatically increased GSH, while having a neutral to negative effect on GSSG, thereby contributing to increasing the GSH ratio in *B. napus* seedlings experiencing Cd stress. The 250 and 500 mg kg^–1^ AsA applications increased GSH 3.62- and 3.16-fold in plant roots and 6.16- and 5.99-fold in plant leaves, respectively, compared to GSH levels in the Cd-only-treated seedlings (Figure 4B). The GSSG level in the 250 mg kg^–1^ AsA-treated seedlings significantly decreased in both plant organs, but the root and leaf GSSG levels in the 500 mg kg^–1^ AsA-treated plants were not significantly different from those of the Cd-only treatment (Figure 4E). AsA treatment significantly increased the GSH redox ratio in both the roots and leaves of Cd-stressed plants (Figure 4H).

Finally, AsA application increased NADPH but had a dose-dependent neutral-to-positive effect on NADP^+^, resulting in neutral (in leaves) to positive (in roots) effects on the NADPH redox ratio. The NADPH levels in the roots and leaves of the 250 and 500 mg kg^–1^ AsA-treated plants greatly increased compared to those of the Cd-only-treated plants (Figure 4C). The NADP^+^ level was not significantly altered in either plant organ in the 250 mg kg^–1^ AsA-treated plants, but it increased in roots and leaves of the 500 mg kg^–1^ AsA-treated plants (Figure 4F). Exogenous AsA treatments significantly increased the NADPH redox ratio in the roots, while not significantly changing it in the leaves (Figure 4I).

### Changes in the Antioxidant Scavenging Enzymes in Cadmium-Stressed Oilseed Rape Seedlings With or Without Ascorbate Application

**FIGURE 5:**
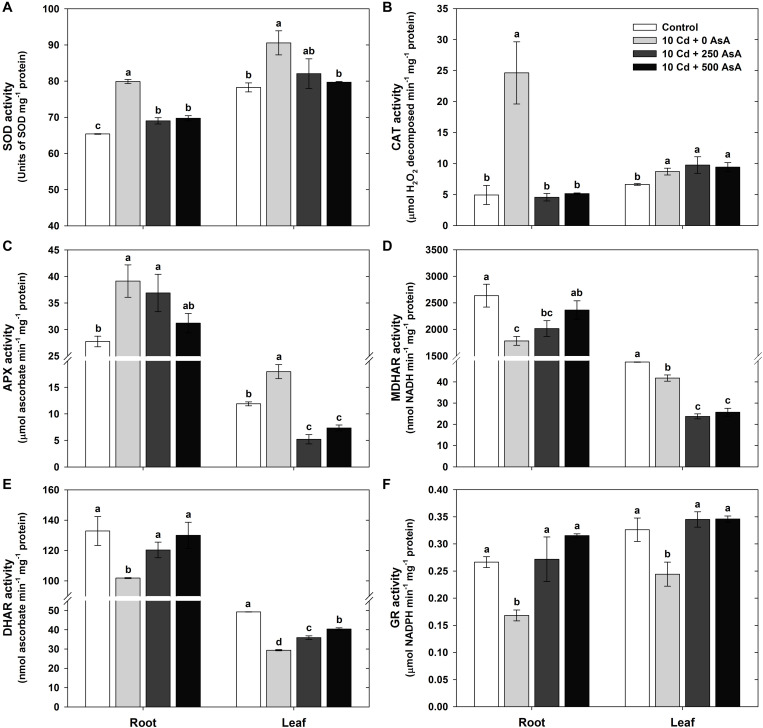
Effect of ascorbate (AsA) application on the activities of antioxidant enzymes of oilseed rape seedlings grown in cadmium (Cd)-treated hydroponics; superoxide dismutase [**(A)** SOD], catalase [**(B)** CAT], ascorbate peroxidase [**(C)** APX], monodehydroascorbate reductase [**(D)** MDHAR], dehydroascorbate reductase [**(E)** DHAR], and glutathione reductase [**(F)** GR] activities. Oilseed rape seedlings at the five-leaf stage were cultivated in a hydroponic solution containing 10 μM Cd and sprayed with AsA (250 and 500 mg kg^–1^). Ten days after treatment, the activity of each antioxidant enzymes in the roots and leaves was measured. The data are presented as mean ± standard deviation (SD) of the mean of three replications (*n* = 3). Means denoted by the same letter are not significantly different at the *P* < 0.05 level according to Fisher’s least significant difference (LSD) test.

With an increase in the amount of AsA applied to Cd-stressed plants, there was a concomitant decrease in the observed amount of Cd-induced ROS (Figure 3). Based on the ROS responses, SOD, CAT, APX, MDHAR, DHAR, and GR activities were measured in the roots and leaves to investigate the influence of AsA on the antioxidant scavenging system in Cd-exposed plants. SOD, CAT, and APX activities in the plants treated with Cd alone increased 1. 22-, 5. 00-, and 1.41-fold in the roots and 1. 16-, 1. 31-, and 1.51-fold in the leaves compared to those of the untreated plants, respectively (Figures 5A–C). However, MDHAR, DHAR, and GR activities were significantly lower in both the roots and leaves under Cd stress (Figures 5D,E).

The Cd-stressed seedlings supplied with either 250 or 500 mg kg^–1^ AsA had significantly lower SOD activity in all organs than that of the seedlings treated with Cd but without AsA (Figure 5A). CAT activity in the roots was decreased by AsA treatment, while no change in CAT activity was observed in the leaves, compared to that of the Cd-only-treated plants (Figure 5B). APX activity decreased in the leaves, but was not significantly altered in the roots, compared to that of the Cd-only-treated plants (Figure 5C). The seedlings treated with Cd + AsA had increased MDHAR activity in the roots, but MDHAR markedly decreased (−43% at 250 mg kg^–1^ AsA, −39% at 500 mg kg^–1^ AsA) in the leaves compared to those of the Cd-treated plants (Figure 5D). Although the Cd alone treatment showed decreases in the DHAR and GR activities, treatment with AsA in the Cd-exposed seedlings resulted in significant increases in these activities in both the roots and leaves compared to those of the Cd alone treatment (Figures 5E,F).

### Heatmap Responses of the Targeted Metabolites of Reactive Oxygen Species and AsA/GSH/NADPH Redox From Cadmium-Stressed Oilseed Rape Seedlings With or Without Ascorbate Application

**FIGURE 6:**
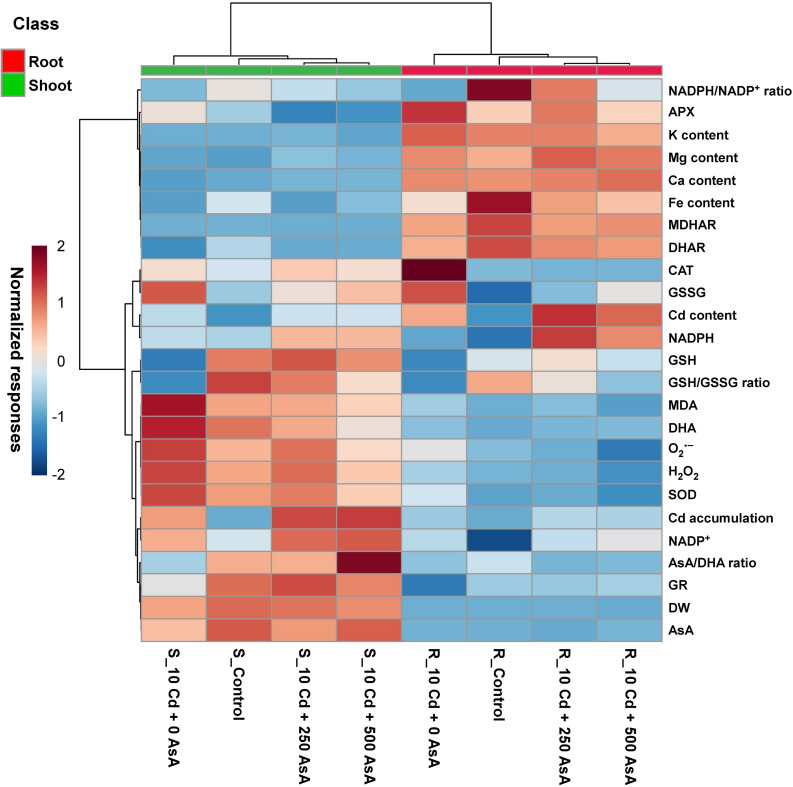
Heatmap analysis of Pearson’s correlation coefficient (*r*) for the targeted metabolites of reactive oxygen species (ROS) and ascorbate (AsA)/glutathione (GSH)/nicotinamide adenine dinucleotide phosphate (NADPH) redox status in cadmium (Cd)-stressed seedlings with or without AsA application. R, root; S, shoot. The numbers after Cd of the code names indicate the AsA level (0, 250, or 500 mg kg^–1^). Red and blue indicate positive and negative normalized responses between the targeted metabolites.

A normalized heatmap matrix system was used to elucidate the overall responses of oilseed rape seedlings to AsA treatment under Cd stress conditions. The results of a normalized heatmap matrix indicated that the treatments elicited differential responses between roots and shoots. In addition, the cluster responses for the Cd-only treatment were the least clustered with the other three treatments irrespective of organ type. That is, AsA treatments were closely clustered with the untreated control in both leaves and roots. Furthermore, the normalized heatmap indicates that, regardless of AsA treatment under Cd stress conditions, plant organs exhibited differential responses across the response variables examined (Figure 6). The normalized responses of GSH, GSH/GSSG ratio, MDA, DHA, ROS, SOD, Cd accumulation, NADP^+^, AsA/DHA ratio, GR, DW, and AsA were relatively higher in shoots than roots. In contrast, NADPH/NADP^+^ ratio, APX, K, Mg, Ca, Fe, MDHAR, DHAR, Cd, and NADPH had relatively higher normalized responses in roots than in shoots (Figure 6).

### Principal Component Analysis of the Targeted Metabolites of Reactive Oxygen Species and AsA/GSH/NADPH Redox From Cadmium-Stressed Oilseed Rape Seedlings With or Without Ascorbate Application

**FIGURE 7:**
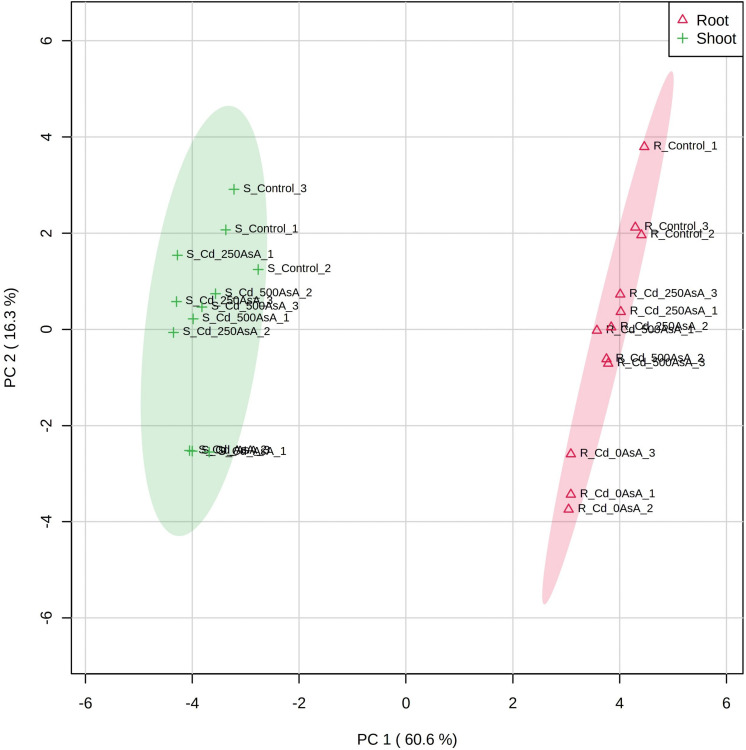
Principal component analysis (PCA) scores plot of the targeted metabolites of reactive oxygen species (ROS) and ascorbate (AsA)/glutathione (GSH)/nicotinamide adenine dinucleotide phosphate (NADPH) redox status in the roots (Δ) and shoots (+) of seedlings treated with different AsA levels. R, root; S, shoot. The numbers after cadmium (Cd) in the code names indicate the AsA level (0, 250, or 500 mg AsA kg^–1^). The last digit indicates which of the three replicates is shown.

A PCA scores plot was applied to evaluate the overall responses of oilseed rape seedlings to AsA treatment under Cd stress conditions. The results of the PCA scores plot accounted for 60.6 and 16.3% of the total variance of principal components (PC) 1 and 2, respectively, and indicated that organ responses to AsA treatment under Cd stress conditions were clearly segregated (Figure 7). Furthermore, the effects of AsA treatment under Cd stress conditions were more apparent in the shoot than in the root (Figure 7). In the PCA, the Cd-only treatment had the most divergent responses from those of the untreated control regardless of organ type. AsA addition treatments appeared closer in the plot to the control than that to the Cd-only treatment (Figure 7).

## Discussion

Many studies have reported that the contamination of agricultural lands by excess Cd adversely affects sustainable crop productivity. Cd toxicity negatively affects photosynthesis by reducing chlorophyll content, which adversely influences general plant growth and development by disrupting vital metabolic processes (Kim et al., 2008; Hong et al., 2013; Jan et al., 2015; Khan et al., 2015; Jung et al., 2016, 2017, 2018b; Farooq et al., 2018). Recently, some studies have demonstrated that the exogenous application of sulfur (Khan et al., 2015; Jung et al., 2017; Tian et al., 2017), glutathione (Chen et al., 2010; Mostofa et al., 2014; Kim et al., 2017; Jung et al., 2019) or ascorbate (Chen et al., 2017; Jung et al., 2018a; Semida et al., 2018; Zhang et al., 2019) may alleviate different heavy metal stresses.

In this study, we have shown that AsA alleviates the heavy metal stress induced by Cd, thereby contributing to reversing the negative effects of Cd on plant growth and development and increasing plant tolerance and accumulation of Cd by ameliorating its phytotoxic effects. For instance, shoot FW and DW of Cd-stressed oilseed rape seedlings were significantly reduced by Cd treatment. However, application of AsA to Cd-exposed oilseed rape seedlings significantly alleviated the negative effects of Cd on the shoot biomass of oilseed rape plants (Table 1). Significantly higher Cd contents were observed in the roots and shoots of Cd-stressed oilseed rape seedlings treated with AsA than in those treated with Cd alone (Figure 1A). Many studies have been reported that Cd uptake and/or accumulation varies by plant species and organs (Chen et al., 2010; Wu et al., 2015; Jung et al., 2016, 2018b). In this study, we revealed that AsA application alleviates the overall phytotoxicity of Cd and increases Cd uptake and accumulation both in roots and shoots of the oilseed rape plant (Figure 1). This shows that AsA promoted Cd accumulation in the roots and increased Cd translocation to the plants because it seems to be critical in reducing the oxidative damage induced by Cd.

Cd exposure induces the production of ROS and a cascade of toxic effects resulting from oxidative stress. As a non-essential heavy metal, Cd can play a key role as a cofactor in Fenton reactions by replacing the essential ions of metalloproteins (Peroza et al., 2009; Singh et al., 2016). The altered reaction modifies essential nutrient uptake (Figure 2) and induces lipid peroxidation in the plant cell *via* the production of ROS, O_2_^⋅–^, H_2_O_2_, and OH⋅, thereby resulting in cell membrane destruction (Figure 3) (Ahmad et al., 2016; Loix et al., 2017). Moreover, it disturbs ion and redox homeostasis in cell metabolic pathways (Gayomba et al., 2013; Asgari Lajayer et al., 2017). This study demonstrated that Cd induced oxidative stresses by the overproduction of O_2_^⋅–^ (Figure 3A) and H_2_O_2_ (Figure 3B) in the roots and shoots of oilseed rape seedlings. This is in agreement with the previous findings of Jung et al. (2017) and Hasanuzzaman et al. (2017).

However, AsA application to Cd-stressed oilseed rape seedlings decreased the level of O_2_^⋅–^ and H_2_O_2_ and thus played a critical role in alleviating the oxidative stress caused by Cd (Figures 3A,B). Increased levels of ROS lead to lipid peroxidation, causing membrane damage and the malfunctioning of ion transporters (Gayomba et al., 2013; Loix et al., 2017). Cd increases the MDA level, which is as an indicator of lipid peroxidation under Cd phytotoxicity (Hasanuzzaman et al., 2017). MDA production in both organs significantly increased in the Cd-stressed seedlings (Figure 3C). However, exogenous application of AsA to Cd-stressed seedlings significantly reduced Cd-induced production of O_2_^⋅–^ (Figure 3A), H_2_O_2_ (Figure 3B), and MDA (Figure 3C) compared to that of Cd alone treatment. AsA-mediated alleviation in oxidative stress is through its antioxidant effect in the non-enzymatic (Figure 4) and enzymatic (Figure 5) antioxidant scavenging systems, thus, leading to declining MDA production, increasing Cd accumulation, and altering the antioxidant defense system in a manner associated with relieving an excessive level of ROS.

In plants, the antioxidants AsA and GSH play a critical role in reducing the oxidative stress induced by heavy metals. Plants can alleviate Cd-induced oxidative stress either enzymatically with antioxidant or redox enzymes or non-enzymatically by employing antioxidant metabolites as electron scavengers (Gill and Tuteja, 2010; Shekhawat et al., 2010; Foyer and Noctor, 2011); both systems for mitigating oxidative stress are essential for maintaining cellular redox homeostasis and in protecting the function of membrane systems (Yan et al., 2015; Hasanuzzaman et al., 2017). AsA plays a critical role in ROS scavenging and redox regulation under heavy metal stress (Chao et al., 2010; Loix et al., 2017; Jung et al., 2018a; Semida et al., 2018; Zhang et al., 2019). GSH is an antioxidant with a sulfhydryl group (-SH) that is important in scavenging the ROS produced by heavy metals (Srivastava et al., 2014; Singh et al., 2015; Kumar and Trivedi, 2018). Therefore, AsA and GSH directly play pivotal roles as antioxidants associated with scavenging ROS and regulating the critical components of the antioxidant defense system (Akram et al., 2017; Kim et al., 2017; Jung et al., 2018a, 2019).

Plant antioxidant content and redox status are therefore informative for understanding plant tolerance to heavy metal stress. For example, it is known that the AsA, GSH, and NADPH levels and their redox ratios are the main factors influencing plant survival under various abiotic stress conditions (Mostofa et al., 2014; Jung et al., 2019). In our study, Cd-exposed plants did not exhibit changes in their NADPH levels, but their AsA and GSH levels significantly decreased in comparison to the untreated plants. Moreover, there was an increase in the levels of DHA, GSSG, and NADP^+^, while the AsA/DHA, GSH/GSSG, and NADPH/NADP^+^ ratios concurrently declined (Figure 4).

Our results are consistent with those of previous studies on the effects of Cd stress on plants and the potential for AsA to alleviate Cd-induced oxidative stress. Cd uptake caused oxidative stress, resulting in increased generation of ROS and lipid peroxidation, as similarly reported in many studies (Gill and Tuteja, 2010; Shekhawat et al., 2010; Foyer and Noctor, 2011; Hasanuzzaman et al., 2017). The present study demonstrates that the oxidative stress induced by Cd in oilseed rape seedlings was correlated further with the redox status of AsA, GSH, and NADPH (Figure 4). However, AsA application improved the AsA, GSH, and NADPH levels and AsA/DHA, GSH/GSSG, and NADPH/NADP^+^ ratios, which might enhance the activities of the AsA–GSH–NADPH cycle to overcome Cd toxicity (Figure 4). Consistent with our results, Chao et al. (2010) and Jung et al. (2018a) reported that exogenous AsA treatment alleviated Cd- and As-induced ROS, lowered lipid peroxidation, increased AsA levels, and helped maintain cellular redox homeostasis in rice seedlings, respectively.

Herein, we have presented an antioxidant mechanism to increase Cd uptake and accumulation in oilseed rape seedlings by alleviating Cd-induced oxidative stress and maintaining the homeostasis of the AsA–GSH–NADPH cycle. Rice seedlings subjected to heavy metal stress exhibited increases in SOD, CAT, and APX activities concomitant with regulation of the enzymes, which are the key antioxidant defense and/or redox regulation pathway leading to synthesis of antioxidants, AsA and GSH and the redox metabolite, NADPH. On the other hand, MDHAR, DHAR, and GR activities often decrease in response to heavy metal exposure (Jung et al., 2018a, 2019). These components in the antioxidant defense system remove ROS, thereby contributing to sustaining the cellular redox status (Sharma et al., 2017).

Our results have demonstrated that relatively low AsA and high DHA levels are maintained by the increase of APX activity or reduction of DHAR activity, thus decreasing the AsA/DHA redox status under Cd-stressed conditions. A low AsA/DHA redox status increases the GSSG level and decreases the GSH/GSSG ratio by regulating DHAR. Under the conditions of Cd stress, GR also plays a critical role in changing the GSH/GSSG ratio by catalyzing the reduction of GSSG to GSH (Figures 4, 5). Simultaneously, GR is downregulated by a reduction in Cd-induced oxidative stress, thereby increasing NADP^+^ content (Chou et al., 2012). This reaction could decrease the NADPH/NADP^+^ redox ratio.

However, applying exogenous AsA to Cd-stressed oilseed rape seedlings markedly decreases the DHA but increases the GSH levels through the downregulation of APX; this increases the AsA/DHA redox state. A high AsA/DHA redox status reduces the GSSG level and enhances the GSH/GSSG ratio by upregulating DHAR. Concomitantly, GR is upregulated by a decrease in Cd-induced oxidative stress with exogenous AsA application, thus increasing the GSH level. This, in turn, increases the GSH/GSSG redox ratio (Figures 4, 5). On the other hand, although NADPH level was increased in Cd-stressed plants with AsA treatment, NADP^+^ also showed high levels of production, so there was no significant change in NADPH/NADP^+^ ratio (Figure 4).

**FIGURE 8:**
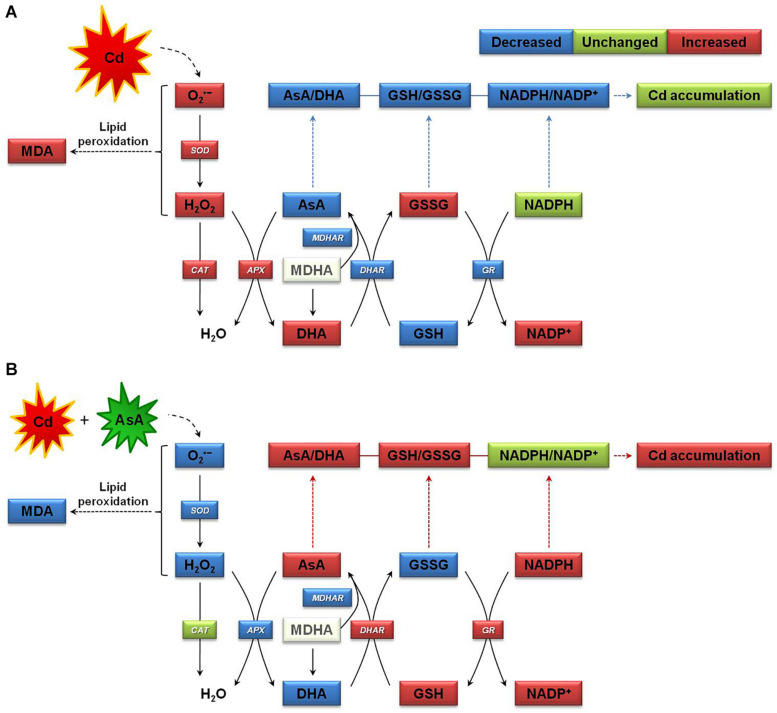
Proposed mechanism of increase in cadmium (Cd) accumulation in oilseed rape seedlings by mitigating Cd-induced oxidative stress and sustaining homeostasis of the ascorbate (AsA)–glutathione (GSH)–nicotinamide adenine dinucleotide phosphate (NADPH) cycle, which involves three interdependent redox couples, AsA/DHA, GSH/GSSG, and NADPH/NADP^+^. **(A)** The Cd-stressed plant growth state and **(B)** the Cd-exposed and AsA-treated plant growth state. APX, ascorbate peroxidase; AsA, ascorbate (reduced form); DHA, dehydroascorbate (oxidized form); AsA/DHA, AsA/DHA redox ratio; CAT, catalase; DHAR, dehydroascorbate reductase; GR, glutathione reductase; GSH, glutathione (reduced form); GSSG, glutathione disulfide (oxidized form); GSH/GSSG, GSH/GSSG redox ratio; MDA, malondialdehyde (a chemical index of lipid peroxidation); MDHA, monodehydroascorbate; MDHAR, monodehydroascorbate reductase; NADPH (reduced form) and NADP^+^ (oxidized form), nicotinamide adenine dinucleotide phosphate; SOD, superoxide dismutase.

Figure 8 provides a simple proposed mechanism for increasing Cd accumulation in oilseed rape plants by alleviating Cd-induced oxidative stress and sustaining homeostasis of the AsA–GSH–NADPH cycle. AsA application to Cd-stressed oilseed rape plants significantly decreased the production of O_2_^⋅–^, H_2_O_2_, and MDA relative to that observed in plants subjected to Cd treatment alone (Figure 8B). As shown here, the proposed mechanism indicates that AsA application to plants under Cd exposure promoted the AsA–GSH–NADPH cycle activities, thereby contributing to increasing Cd accumulation in the plants and improving the ROS scavenging system, which increased AsA-induced Cd resistance in oilseed rape plants.

## Conclusion

Our results clearly indicate that exogenous AsA contributes to the alleviation of Cd-induced oxidative stress in oilseed rape plants by a concomitant promotion of the components of the antioxidant scavenging defense system. Our results also demonstrate that exogenous AsA application to oilseed rape seedlings under Cd stress significantly alleviated the overall Cd toxicity by regulating the homeostasis of the AsA–GSH–NADPH cycle, which reestablishes the cellular redox status. However, large-scale field experiments should be highly valuable in developing a phytoremediation and/or sustainable agriculture production system for especially Cd-contaminated lands. Therefore, the results reported here provide invaluable insights into the critical role of AsA in regulating the AsA–GSH–NADPH cycle system and, as a result, the Cd uptake, accumulation, and resistance of oilseed rape plants.

## Data Availability Statement

The original contributions presented in the study are included in the article/supplementary material, further inquiries can be directed to the corresponding author.

## Author Contributions

HJ designed the experiment. HJ and B-RL performed the experiment. HJ, B-RL, M-JC, E-JL, T-GL, G-BJ, M-SK, and JL analyzed the data and provided constructive comments and discussion on the manuscript. HJ and JL wrote, revised, and corrected the manuscript. All authors contributed to the article and approved the submitted version.

## Conflict of Interest

The authors declare that the research was conducted in the absence of any commercial or financial relationships that could be construed as a potential conflict of interest.

## Footnotes

1 www.metaboanalyst.ca
